# Upregulated synthesis and production of bioactive compounds in *Lotus arabicus* L. by in vitro feeding with dried powder of date palm seeds

**DOI:** 10.1186/s12870-024-04919-7

**Published:** 2024-03-27

**Authors:** Doaa E. Elsherif, Fatmah A. Safhi, Asmaa M. Khalifa, Gehad A. Ragab

**Affiliations:** 1 Botany Department, Faculty of Science, Tanta University, Tanta, 31527 Egypt; 2 Department of Biology, College of Science, Princess Nourah Bint Abdulrahman University, P.O. Box 84428, Riyadh, 11671 Saudi Arabia; 3 Botany and Microbiology Department, Faculty of Science, Al Azhar University (Girls Branch), Cairo, Egypt

**Keywords:** Callus culture, Date palm seeds, Elicitation, Flavonoids, *Lotus arabicus*, Phenolics

## Abstract

**Background:**

Plants are considered the primary source of many principal bioactive compounds that have been utilized in a wide range of applications including the pharmaceutical and biotechnological industries. Therefore, there is an imperative need to modulate the production of natural bioactive components. The present study aimed to determine the importance of dried and pulverized date palm seeds (DPS) as a natural elicitor for the synthesis of secondary metabolites in *Lotus arabicus* L.

**Results:**

The presence of various antioxidant compounds, simple sugars, amino acids, fatty acids and reasonable mineral contents was distinct in the phytochemical characterization of DPS. The major components detected in DPS analysis were the 5-(hydroxymethyl) furfural and 2,3-dihydro-3,5-dihydroxy-6-methyl-4H-pyranone. The induced callus of *L. arabicus* (seven weeks old) was supplemented with DPS at different concentrations (0, 2, 4, 8 and 10 g/l) in culture media. Treatment with 8 g/l DPS induced the highest antioxidant capacity, ascorbic acid content and secondary metabolites (total phenolics and flavonoids) in the produced callus. Stress biomarkers (hydrogen peroxide and malondialdehyde) were found in the control ranges except at 10 g/l DPS. The expression patterns of key genes involoved in secondary metabolism modulation, such as phenylalanine ammonia lyase (*PAL*), chalcone synthase (*CHS*), chalcone isomerase (*CHI*), flavonol synthase (*FLS*) and deoxyxylulose phosphate reductoisomerase (*DXR*), were triggered after DPS treatments. Moreover, the quantitative profiling of phenolic and flavonoid compounds showed that supplementation with DPS, especially at 8 g/l, led to pronounced increases in most of the measured compounds.

**Conclusion:**

The marked upregulation of eliciting-responsive genes and overproduction of secondary metabolites provide molecular-based evidence for intensifying the principal pathways of phenylpropanoid, flavonoid and terpenoid biosynthesis. Overall, the present in vitro study highlights the stimulating capacity of DPS utilization to improve the bioactive components of *L. arabicus* at the physiological and molecular levels, enhancing its potential as a medicinal herb.

**Supplementary Information:**

The online version contains supplementary material available at 10.1186/s12870-024-04919-7.

## Background

*Lotus arabicus* L. is a wild annual herb that belongs to the Fabaceae family. It has been traditionally used in folk medicine and in the production of pharmaceuticals and therapeutics due to its bioactive compounds including alkaloids, phenolics, flavonoids, terpenoids, tannins and cardiac glycosides [1]. The positive antimicrobial activity of *Lotus arabicus* L. was significantly associated with its rich bioactive components, which included tannins, coumarins and flavonoids [2]. Plant tissue cultures can serve as factories of secondary metabolites with the benefit of consistency in yield and quality [3]. The content of secondary metabolites in tissue cultures could be boosted using various methods, such as optimizing the composition and conditions of the culture medium and feeding with precursors and elicitors.

Secondary metabolites play a distinct role in plant protection from several stresses, which are often categorized into three major classes: phenolics, terpenoids and alkaloids [4, 5]. As the largest group of secondary metabolites, polyphenols exist in all plant species and play various biological roles in defense mechanisms [6]. Polyphenols are phenylpropanoids that are primarily synthesized from phenylalanine and are classified into many groups, such as phenolic acids, flavonoids, tannins, stilbenes and lignans [7]. The phenylpropanoid metabolic pathway involves the conversion of phenylalanine to p-coumaroyl-CoA by a three-step enzymatic reaction started by phenylalanine ammonia-lyase (PAL) [8]. Further enzymatic reactions involving chalcone synthase (CHS) and chalcone isomerase (CHI) result in the synthesis of different classes of flavonoids [9]. Moreover, flavonol synthase (FLS) plays a central role in the production of flavonols from dihydroflavonols [10]. Each step in this pathway is essential for the biosynthesis of a wide range of flavonoid compounds with distinct chemical structures and biological activities.

Terpenoids, as naturally occurring phytochemicals, contribute to normal plant growth and development and play crucial roles in plant interactions with environmental stresses [11]. They include diverse classes of primary and secondary metabolites such as phytohormones, photosynthetic pigments and essential oils [12, 13]. It was reported that *L. arabicus* L. contains high levels of the terpenoid sitosterol, which has many health benefits [1]. Most terpenoid derivatives are synthesized via the mevalonate (MVA) pathway and plastid-localized methylerythritol phosphate (MEP) pathway [14]. In the enzymatic chloroplast pathway, a main upstream enzyme, deoxyxylulose phosphate reductoisomerase (DXR), is known to catalyze the reduction of deoxyxylulose phosphate (DXP) to MEP, which is oxidized to isopentenyl pyrophosphate (IPP), the common precursor of terpenoid biosynthesis. DXR activation has been reported to promote terpenoid pathways as a key regulatory enzyme controlling their synthesis [13, 15].

To increase the production of bioactive compounds, natural bioproducts have been employed as elicitors in plant tissue culture. Therefore, the availability of sustainable applications enhancing secondary metabolism productivity should be evaluated. The seeds of date palm (*Phoenix dactylifera*) are considered byproducts that are massively disposed of daily, particularly in Middle Eastern countries. The costless seeds of date palm plants are a source of many components with high nutritional value such as oil, fibers, minerals, proteins, amino acids, fatty acids and phenolic compounds [16]. The date seeds exhibited high antioxidant activity when compared to standard antioxidants due to their phenolic compounds such as naringenin and rutin [17]. The rich contents make date palm seeds (DPS) a convenient organic application for eliciting plant secondary metabolism and growth. Despite that fact, this study is mostly the first to explore the convenience of DPS in plant tissue culture applications. Therefore, the objective of this investigation is to highlight the usage of date palm dried seeds as an innovative plant tissue culture application for provoking synthetic pathways of valuable secondary metabolites in *L. arabicus* L. callus.

## Materials and methods

### Preparation and phytochemical analysis of date palm seeds

The dry fruits of date palm (*Phoenix dactylifera*) were purchased from a local market (the common name is Aswani). The fruit pulp was removed to obtain the dry date seeds (kernels). The seeds were washed twice in distilled water, air-dried, crushed into a fine powder using a mill and passed through a sieve 2 mm in length.

For the qualitative characterization, a definite weight of DPS was soaked in 90% ethanol to extract its constituents and subsequently filtered to obtain the ethanolic extract, which further subjected to gas chromatography-mass spectrometry (GC‒MS) analysis [18] and phytochemical analyses. The ethanolic extract (1 µl) was introduced to the GC mass spectrometer (Perkin Elmer model: clarus 580/560S) capillary column (Elite-5MS, 30 m 0.25 mm ID 0.25 µm df) using an autosampler AS3000 coupled with GC in split mode. The initial temperature of the oven was 60 °C, then increased to 210 °C at a 5 °C/min rate with a 6 min hold, and subsequently increased at a rate of 10 °C/min to 280 °C. The carrier gas (helium) was applied in constant pressure mode at a flow rate of 1 ml/min. The chemical constituents of DPS were characterized according to the GC retention time (RT). The resultant mass spectra were computer-matched with those of standard compounds (50 to 620 Da) based on laboratory reference libraries from the NIST and Pfleger databases.

The mixed acid–digestion method was employed for phytochemical analysis. Approximately 0.5 g of DPS was digested by mixing with a HNO_3_:H_2_O_2_ mixture (5:3, v/v) and gently heated until a clear solution was obtained [19]. The solution was then diluted to a constant volume, filtered and further subjected to mineral analysis. The levels of K, Ca and Mg were estimated using inductively coupled plasma-optical spectroscopy (Polyscan 61E, Thermo Jarrell-Ash Corp., Franklin, MA, USA). A colorimetric assay with the Rochelle reagent was used to estimate the N ion content, and the molybdenum blue technique was utilized to detect the P ion content against standard calibration curves, according to [20]. Furthermore, the phenolic and flavonoid contents and total antioxidant activity were measured in the ethanolic extract of DPS.

###  Callus induction and in vitro culture of *L. arabicus*

**Fig. 1:**
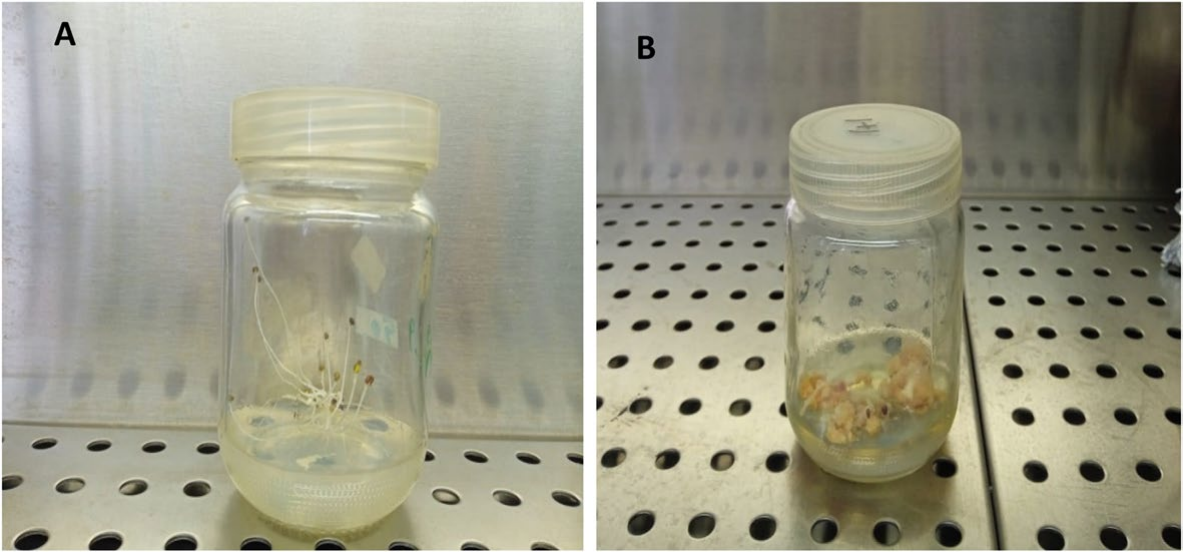
**A** The seeds of *L. arabicus* were germinated on half of the Murashige and Skoog (MS) media. **B** Callus was induced from stem explant MS media supplemented with 1 mg/l 2,4-D and 1 mg/l BA

The seeds of *L. arabicus* were collected from Al-Azhar Gardens (30.0408°N, 31.2647°E), Egypt. They were surface sterilized by dipping in 70% ethanol for 1 min, immersed in a 50% Clorox solution containing 5.4% sodium hypochlorite (NaOCl) for 20 min, and subsequently rinsed five times with sterilized distilled water. Surface sterilization of the seeds was carried out under complete aseptic conditions in a laminar air flow hood. The sterilized seeds were aseptically air-dried and germinated on half-strength Murashige and Skoog (MS) media [21] supplemented with 30 g/l sucrose and 10 g/l agar. Cultures were incubated at 25 ± 2°C in the dark to enable germination. The clean and sterilized stem explants obtained from the germinated seeds were cut into 1.5 cm segments and inoculated on solid MS media supplemented with 1 mg/l 2,4-D (2,4-dichlorophenoxy acetic acid) and 1 mg/l BA (6-benzyladenine) for callus induction (Fig. 1).

After seven weeks, the successfully induced calli were separated from the explants and cultured separately until a sufficient amount of callus was produced. The obtained calli were grown on MS media supplemented with 1 mg/l, 2,4-D and 1 mg/l BA; treated with different concentrations of DPS powder (2, 4, 8 and 10 g/l) and then incubated at 25 ± 2°C in the dark. After seven weeks, the calli were separated from the media for further measurements.

### Evaluation of growth behavior and secondary metabolites

After seven weeks, the calli were collected and the fresh weights were measured. Thereafter, the fresh samples were dried at 40°C for 48 h and then used to evaluate dry weight. Total phenolic and flavonoid levels were detected in the ethanolic extract of callus samples according to [22, 23], respectively. To estimate the total phenolics, the sample ethanolic extract was incubated with Folin − Ciocalteu’s reagent and 20% Na_2_CO_3_ for 1 h, where the absorbance was measured against standard phenol at 650 nm. The content of total flavonoids was quantified at 417 nm in a mixture containing sample extract, 10% AlCl_3_, 1 M K − acetate and 95% ethanol against a standard of quercetin.

### Total antioxidant capacity (TAC)

The phosphomolybdate method was used to determine the total antioxidant capacity of the ethanolic extracts, employing ascorbic acid (ASA) as a standard [24]. A 0.3 ml aliquot of sample extract was blended with 3 ml of reagent solution (0.6 M sulfuric acid, 28 mM sodium phosphate and 4 mM ammonium molybdate). The tubes were incubated in a water bath at 95 °C for 90 min. Afterwards, the tubes were left to cool to room temperature, and the absorbance was measured at 695 nm against a blank of ASA. The TAC was expressed as mg/g ASA equivalent.

### Determination of ascorbic acid

Ascorbic acid, as a non-enzymatic antioxidant, was estimated according to [25]. Callus tissues were extracted with 5% sulfosalicylic acid. The reaction mixture contained 2% Na molybdate, 0.15 N H_2_SO_4_, 1.5 mM Na_2_HPO_4_ and callus extract. The mixture was incubated at 60 °C in a water bath for 40 min and then immediately cooled. The absorbance was measured at 660 nm, and the ascorbic acid content was calculated as mg/g fresh mass (FM).

### Evaluation of stress biomarkers

The contents of hydrogen peroxide (H_2_O_2_) and malondialdehyde (MDA) were assessed according to [26, 27], respectively. Fresh calli (0.1 g) were extracted in 0.1% trichloroacetic acid to estimate H_2_O_2_ concentration. The callus extract was mixed with phosphate buffer (pH 7.0) and KI_2_ (1 M), and the absorbance was read at 390 nm. The level of H_2_O_2_ was calculated with the aid of a coefficient of 0.28 µM^–1^ cm^–1^. For MDA detection, as a product of lipid peroxidation, callus samples were extracted in 5% (w/v) trichloroacetic acid. The reaction mixture consisted of sample extract and 0.67% (w/v) TBA; the mixture was boiled for 20 min and then immediately cooled. The absorbance was read at 532 and 600 nm, where the extinction coefficient (155 mM^−1^ cm^−1^) was utilized to measure the MDA concentration.

### Quantitation of relative expression levels of the specific key genes

Fresh *L. arabicus* callus tissues were pulverized in liquid nitrogen to extract total RNA using an RNeasy Mini Kit (Qiagen). To obtain complementary DNA (cDNA), a reverse RNA transcription reaction was performed using Invitrogen’s superscript III Reverse Transcriptase Kit in a thermocycler (Inc., PTC-100™ Programmable Thermal Controller, USA).

The expression patterns of specific key genes involved in secondary metabolism were analyzed via quantitative real-time polymerase chain reaction (qRT‒PCR) using a Rotor-Gene 6000 (QIAGEN, Q 5 PLEX System, Germany). The reaction mixture contained SYBR Green PCR Master Mix (Fermentas, USA) and specific primer pairs, as described in Table S1. The gene transcript levels were normalized to those of the *GAPDH* reference gene. The initiation step in the amplification program was adjusted to 95 °C for 10 min for denaturation, followed by 40 cycles of 95 °C for 15 s, annealing at 60 °C for 30 s and extension at 72 °C for 30 s. All sample manipulations were performed in triplicate. The quantification of relative transcripts and expression levels of the studied genes was performed during the extension phase using the 2 ^−−∆∆CT^ method [28].

### Quantitative analyses of phenolic acids and flavonoids

The ethanol extract of *L. arabicus* was prepared for use in high-performance liquid chromatography (HPLC) analysis using an Agilent 1260 series [29]. The standard compound mixtures were utilized for quantification including phenolic compounds (rosmarinic acid, caffeic acid, syringic acid, coumaric acid, vanillin, ferulic acid, chlorogenic acid and cinnamic acid) and flavonoids (daidzein, catechin, rutin, naringenin, quercetin and kaempferol). The HPLC chromtogram for the standard mixture is shown in Fig. S3. The separation was carried out using an Eclipse C18 column (4.6 mm × 250 mm i.d., 5 μm) for flavonoid and phenolic compound analysis. The separation of phenolic acids and flavonoids was carried out with a mobile phase consisting of water (A) and 0.05% trifluoroacetic acid in acetonitrile (B) at a flow rate of 0.9 ml/min. The mobile phase was programmed consecutively in a linear gradient as follows: 0 min (82% A); 0–5 min (80% A); 5–8 min (60% A); 8–12 min (60% A); 12–15 min (82% A); 15–16 min (82% A); and 16–20 (82%A). The multi-wavelength detector was monitored at 280 nm. The injection volume was 5 μl for each of the sample solutions. The column temperature was maintained at 40 °C.

### Statistical analysis

The results are presented as the mean of three replicates ± standard error (SE). Differences between treatments for the different measured variables were tested by one-way variance (ANOVA), followed by Duncan’s test with significant differences found (*p* < 0.05) using XLSTAT software (version 2014.5.03). The Pearson correlation coefficient and principle component analysis (PCA) between different parameters and different DPS treatments were performed using XLSTAT software.

## Results

### Qualitative GC‒MS analysis of DPS

The major phytochemical components, identified via GC analysis, are listed in Table 1 according to the obtained chromatogram (Fig. S1). At RT of 12.726 min, 5-(hydroxymethyl)furfural (HMF) was detected as the most abundant compound. The second highest component was 2,3-dihydro-3,5-dihydroxy-6-methyl-4H-pyranone (DDMP), which was observed at RT of 10.625 min. The 2-furanone, 5-heptyldihydro and palmitic acid were detected as the third-highest compounds at RT of 17.043 and 24.416 min, respectively. Furthermore, 3-deoxy-d-mannoic lactone and phenol 2,5-bis(1,1-dimethylethyl were detected at RT of 19.344 and 17.798 min, respectively. The D-alanine N-propargyloxycarbonyl- isohexyl ester and 2-ethylcyclohexyl ester were identified at RT of 8.324 and 12.481 min, respectively. Alternatively, several fatty acids, including palmitic acid, octadecanoic acid, myristic acid, pentanoic acid and linoleic acid, were recorded with low ratios at RT of 24.416, 27.922, 21.324 and 24.035, respectively. Furthermore, tiny amounts of fatty alcohols (tridecan-1-ol and hexadecanol) and oxalic acid were observed.

**Table 1:** Phytochemical constituents identified in DPS by GC–MS

No	**Area %**	Compounds	RT	RI	**Height**	**Molecular formula**
1	20.914	5-(Hydroxymethyl) furfural	12.726	1233.2	108,709,800	C_6_H_6_O_3_
2	12.571	2,3-Dihydro-3,5-dihydroxy-6-methyl-4H-Pyran-4-one	10.625	1154.4	26,333,422	C_6_H_6_O_4_
3	7.761	2(3H)-Furanone, 5-heptyldihydro-	17.043	1574	6,687,944	C_11_H_20_O_2_
4	7.549	Palmitic acid	24.416	1962	34,546,876	C_16_H_32_O_2_
5	3.307	3-Deoxy-d-mannoic lactone	19.344	1667	4,305,016	C_6_H_10_O_5_
6	1.974	D-Alanine, N-propargyloxycarbonyl-, isohexyl ester	8.324	1725	4,880,929	C_13_H_21_NO_4_
7	1.517	Pentanoic acid, 2-methyl-, ethyl ester	12.481	936	5,017,558	C_8_H_16_O_2_
8	1.345	Phenol, 2,5-bis(1,1-dimethylethyl	17.798	1517	1,585,498	C_14_H_22_O
9	1.311	Octadecanoic acid	27.922	2200	289,267.0	C_18_H_36_O_2_
10	1.203	Myristic acid	21.324	1763	265,465.2	C_14_H_28_O_2_
11	1.062	Tridecan-1-ol	27.477	1575	234,357.7	C_13_H_28_O
13	0.969	Oxalic acid	19.249	748	4,435,632	C_2_H_2_O_4_
14	0.895	Linoleic acid	24.035	2145.8	4,140,784	C_18_H_32_O_2_
15	0.808	Hexadecanol	23.095	1880	178,167.7	C_16_H_34_O

*RT* Retention time, *RI* Retention index

### Phytochemical characterization of DPS

The DPS was phytochemically characterized to screen its components and interpret their promoting impact, as shown in Table S2. The mineral composition of DPS exhibited the highest P content, with 8.3 mg/g dry mass (DM). The level of N was 4 mg/g DM, while the contents of K and Ca were similarly represented (approximately 1 mg/g DM). The level of Mg ions was the lowest at 0.2 mg/g DM. On the other hand, DPS analysis revealed marked antioxidant activity (20.8 mg ASA/g DM), demonstrating the high availability of antioxidants. In this context, phenolic compounds were highly detected at 45.5 mg/g DM, and total flavonoid compounds were detected at a convenient level.

### Biomass and secondary metabolites assessment

**Fig. 2:**
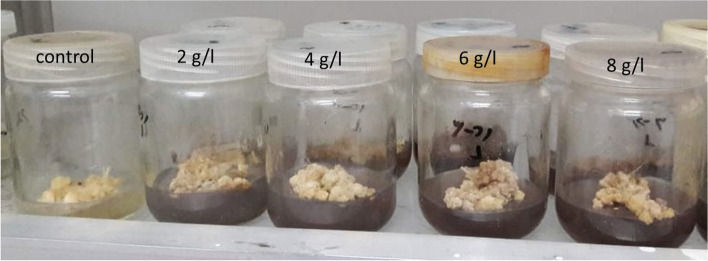
Effect of different concentrations of date palm seed powder (2, 4, 8 and 10 g/l) on *L. arabicus* L callus

**Fig. 3:**
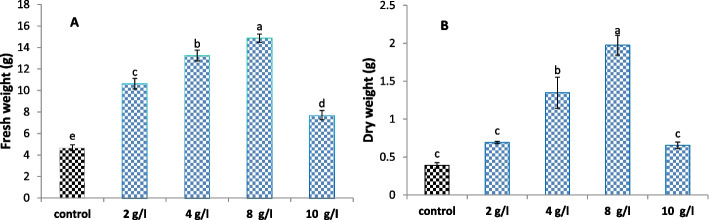
**A** Fresh biomass and (**B**) dry biomass of *L. arabicus* L callus grown under different concentrations of DPS (2, 4, 8 and 10 g/l). The data are means ± SE. Different small letters indicate statistically significant differences between different treatments according to Duncan’s test (*P* < 0.05)

As shown in Figs. 2 and 3, treatment of *L. arabicus* callus with DPS had an enhanced effect on biomass yield. The serial sets of DPS powder caused a gradual increase in fresh and dry biomasses, while 8 g/l DPS exhibited the most pronounced improvement by 218% and 406%, respectively compared to those of the control.

**Fig. 4:**
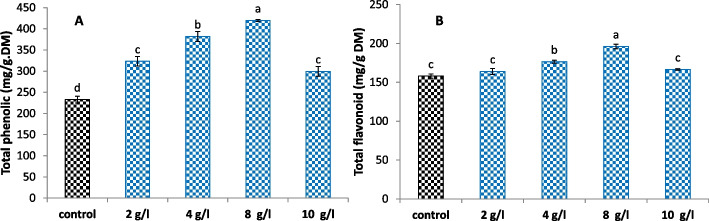
**A** total phenolic content and (**B**) total flavonoid content of *L. arabicus* L callus grown under different concentrations of DPS (2, 4, 8 and 10 g/l). The data are means ± SE. Different small letters indicate statistically significant differences between different treatments according to Dunnett’s test (*P* < 0.05)

On the other hand, the content of total phenolics and total flavonoids, as secondary metabolic markers, were determined as shown in Fig. 4. The results indicated that, compared with those in the control, the DPS-supplemented calli in the treatment group had significantly elevated levels of total phenolics in a dose-dependent manner, with a maximum increase of 80% occurring at 8 g/l DPS. Regarding the total flavonoid content, both the 4 and 8 g/l DPS treatments exhibited significant increase percentages of 12% and 24%, respectively, in comparison to the control.

### Antioxidant capacity and stress biomarkers detection

**Fig. 5:**
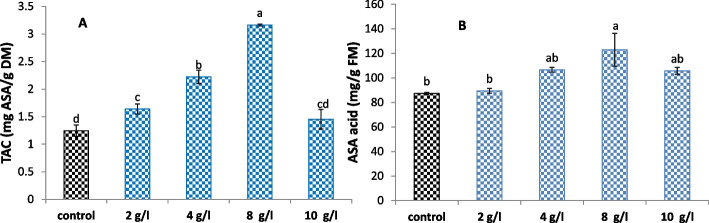
**A** Total antioxidant capacity and (**B**) ascorbic acid, of *L. arabicus* L callus grown under different concentrations of DPS (2, 4, 8 and 10 g/l). The data are means ± SE. Different small letters indicate statistically significant differences between different treatments according to Duncan’s test (*P* < 0.05)

The obtained results shown in Fig. 5 indicate that the addition of DPS doses to the culture media significantly elevated the TAC and ASA contents when compared to their counterparts in the control callus tissues. Maximum increases of 154% and 41% in TAC and ASA, respectively, were recorded in 8 g/l DPS-treated calli relative to the control. In addition, supplementation with DPS in the culture media significantly increased the ASA content as a sign of enhanced TAC compared to the control treatment. Callus treated with 10 g/l DPS showed a non-significant increase of 17% in TAC in comparison with the control.

**Fig. 6:**
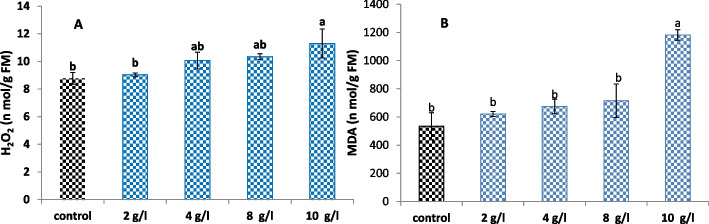
**A** H_2_O_2_ and (**B**) MDA of *L. arabicus* L callus grown under different concentrations of DPS (2, 4, 8 and 10 g/l). The data are means ± SE. Different small letters indicate statistically significant differences between different treatments according to Duncan’s test (*P* < 0.05)

Stress marker (MDA and H_2_O_2_) contents were determined to investigate whether the application of DPS generates oxidative stress in treated *L. arabicus* calli, as shown in Fig. 6. Currently, the high level of DPS (10 g/l) exhibited significant increases in MDA and H_2_O_2_ contents by 153% and 29%, respectively, in comparison to the control. However, lower DPS doses (2, 4 and 8 g/l) caused non-significant changes in MDA and H_2_O_2_ contents, matching control levels.

### Modifications of the expression level of genes in *L. arabicus* callus

**Fig. 7:**
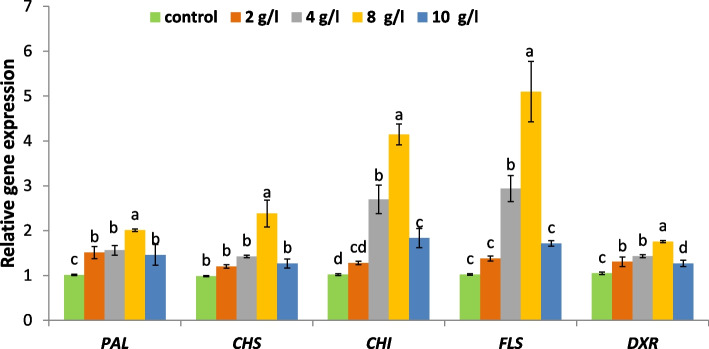
Relative gene expressions of *CHS, CHI, FLS, PAL* and *DXR* in *L. arabicus* L callus. Data are means of three replicates ± SE. Different small letters indicate statistically significant differences between different treatments according to Duncan,s test (*P* < 0.05)

Changes in the expression levels of key genes involved in secondary metabolite biosynthetic pathways were also examined. The relative gene expression levels of *PAL, CHS, CHI, FLS* and *DXR* were determined to explore the eliciting role of DPS levels, as shown in Fig. 7. The application of DPS different sets markedly elevated the expression of these genes, indicating higher transcript levels comparable to the control. The moderate dose of DPS extract (8 g/l) was responsible for the highest increase in *PAL, CHS, CHI, FLS* and *DXR* expression by 2.0-, 2.4-, 4.0-, 4.9- and 1.6-fold, respectively, compared with the nontreated control.

### Quantitative analyses of phenolic acids and flavonoids

HPLC was employed to quantitatively differentiate the major phenolic and flavonoid components in DPS-treated *L. arabicus* calli (Table 2, Fig. S2). The results revealed eight phenolic compounds (rosmarinic acid, caffeic acid, syringic acid, coumaric acid, vanillin, ferulic acid, chlorogenic acid and cinnamic acid) and six flavonoids (daidzein, catechin, rutin, naringenin, quercetin and kaempferol). The results reflected a highly increased concentration of phenol and flavonoid constituents under DPS treatment, especially at 8 g/l, which was 145% greater than that of the untreated control. Compared to those of the control, considerable amounts of cinnamic acid derivatives, such as chlorogenic acid (15.2-fold) and rosmarinic acid (2.2-fold) were obtained at 8 g/l DPS, while the maximum caffeic acid level (4-fold) was recorded at 4 g/l DPS. In addition, significant quantities of the benzoic acid derivative vanillin and the cinnamic acid derivative ferulic acid were highly accumulated in 8 g/l DPS-treated calli.

**Table 2:** Quantitative determination of phenolic acids and flavonoid compounds of *L. arabicus* L. callus treated with DPS (0, 2, 4, 8, 10 g/l) by using high-performance liquid chromatography (HPLC)

No	Category	Compounds	0	2 g/l	4 g/l	8 g/l	10 g/l	RT
			µg/ml					min
1	**Phenolic**	Rosmarinic acid	0.45 ± 0.02^e^	0.63 ± 0.01^d^	1.24 ± 0.02^b^	1.43 ± 0.04^a^	0.85 ± 0.03^c^	11.9
2		Caffeic acid	1.38 ± 0.8^d^	2.17 ± 0.3^cd^	5.41 ± 0.35^a^	4.04 ± 0.67^b^	2.43 ± 0.25^c^	6.09
3		Syringic acid	0.27 ± 0.01^ab^	0.18 ± 0.01^cd^	0.34 ± 0.03^a^	0.22 ± 0.08^bc^	0.11 ± 0.02^d^	6.49
4		Coumaric acid	0.07 ± 0.01^b^	0.23 ± 0.06^a^	0.11 ± 0.03^b^	0.19 ± 0.04^a^	0.21 ± 0.03^a^	8.90
5		Vanillin	0.23 ± 0.05^d^	0.49 ± 0.01^c^	0.75 ± 0.03^b^	0.97 ± 0.07^a^	0.74 ± 0.02^b^	9.76
6		Ferulic acid	0.07 ± 0.01^b^	ND	0.07 ± 0.01^b^	0.09 ± 0.02^a^	0.10 ± 0.01^a^	10.0
7		Chlorogenic acid	6.14 ± 0.34^c^	13.76 ± 0.5^b^	15.59 ± 1.8^b^	21.39 ± 2.3^a^	15.7 ± 1.5^b^	4.15
8		Cinnamic acid	0.12 ± 0.01^c^	0.14 ± 0.04^bc^	0.18 ± 0.05^abc^	0.21 ± 0.02^a^	0.19 ± 0.04^ab^	19.3
9	**Flavonoids**	Catechin	0.67 ± 0.1^b^	1.13 ± 0.8^ab^	3.05 ± 1.9^a^	3.12 ± 1.5^a^	1.28 ± 0.9^ab^	4.62
10		Rutin	0.62 ± 0.2^ab^	0.68 ± 0.1^ab^	0.70 ± 0.14^ab^	0.79 ± 0.08^a^	0.52 ± 0.08^b^	6.8
11		Naringenin	0.13 ± 0.05^b^	0.33 ± 0.06^b^	1.59 ± 0.06^a^	1.89 ± 0.9^a^	2.43 ± 0.7^a^	10.29
12		Quercetin	0.57 ± 0.05^d^	0.33 ± 0.04^c^	0.56 ± 0.01^c^	0.88 ± 0.03^a^	0.76 ± 0.08^b^	17.12
13		Kaempferol	1.62 ± 0.6^a^	ND	0.36 ± 0.03^c^	0.97 ± 0.05^b^	1.15 ± 0.2^ab^	20.3
14		Daidzein	0.13 ± 0.03^b^	0.11 ± 0.04^b^	0.24 ± 0.01^a^	0.09 ± 0.01^b^	0.21 ± 0.08^a^	16.07
**Total content**			20.18 ± 1.1^d^	19.07 ± 0.6^c^	30.19 ± 2.4^b^	36.28 ± 0.3^a^	26.68 ± 2.14^b^	

The data are means ± SE. Different small letters indicate statistically significant differences between different treatments according to Duncan’s test (*P* < 0.05)

Among the flavonoid constituents, 8 g/l DPS had the highest concentrations of catechin, quercetin rutin by 2.4-, 0.31- and 0.17-fold greater than those of the control, respectively. The naringenin level peaked at 10 g/l DPS by 2.3-fold compared to the control. Conversely, the DPS-recommended dose (8 g/l) caused a significant reduction in kaempferol content relative to the control.

### Pearson correlation coefficient

**Fig. 8:**
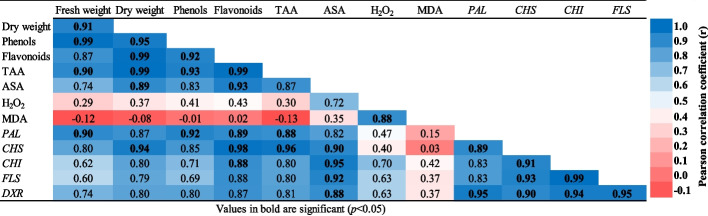
Pearson correlation of physiological parameters and gene expressions level for secondary metabolites pathway measured at control and different DPS concentrations of *L. arabicus* L callus (2, 4, 8 and 10 g/l)

Pearson’s simple correlation was applied to discern the relationships between the physiological and molecular attributes recorded in different treatments with DPS application (Fig. 8). The results indicated that the phenolic and flavonoid contents and total antioxidant capacity were strongly correlated with each other and positively correlated with the increased expression levels of the *PAL, CHS, CHI* and *FLS* genes. In addition, the ascorbic acid content was positively correlated with flavonoid, *CHS, CHI and FLS* expression levels. Furthermore, dry weight was significantly correlated with phenolic, flavonoid, TAC and ASA contents. On the other hand, the MDA content was negatively correlated with fresh and dry callus biomasses, secondary metabolites (phenolics and flavonoids) and TAC.

### Principal component analysis

**Fig. 9:**
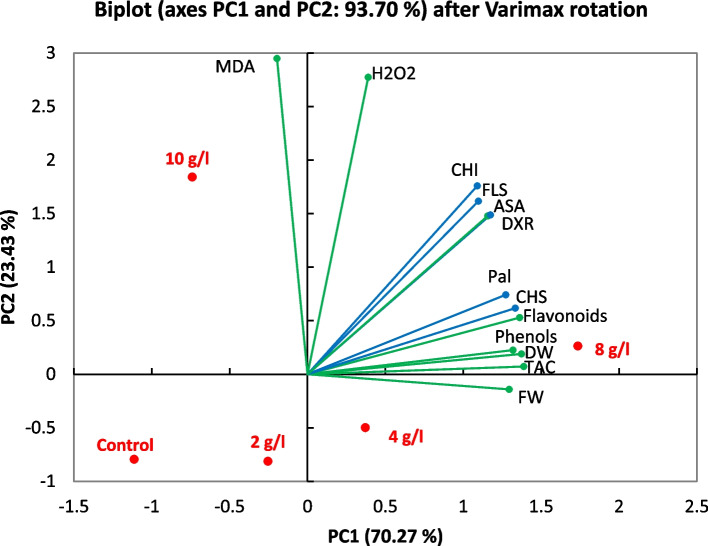
Principal component analysis of physiological parameters and gene expression levels for secondary metabolites pathway measured at the control and different DPS concentrations of *L. arabicus* L callus (2, 4, 8 and 10 g/l DPS)

The principal component analysis results interpreted DPS treatments with physiological attributes and relative expressions of studied genes (Fig. 9). The first four components (PCs) accounted for 100% of the total variation, with the first three PCs with eigenvalues ≥ 1 explaining 93.70% of the total variation. The first PC accounted for 70.27% of the total variation in the data that was significantly correlated with flavonoids, *CHS*, ASA, dry weight, *DXR*, TAC, *PAL, CHI, FLS*, phenols and fresh weight, in descending order. PC2 explained 23.43% of the total variation and was mainly influenced mainly by MDA and H_2_O_2_. In the PCA biplot of the first two PCs, which accounted for 70.27% of the overall variance.

**Fig. 10:**
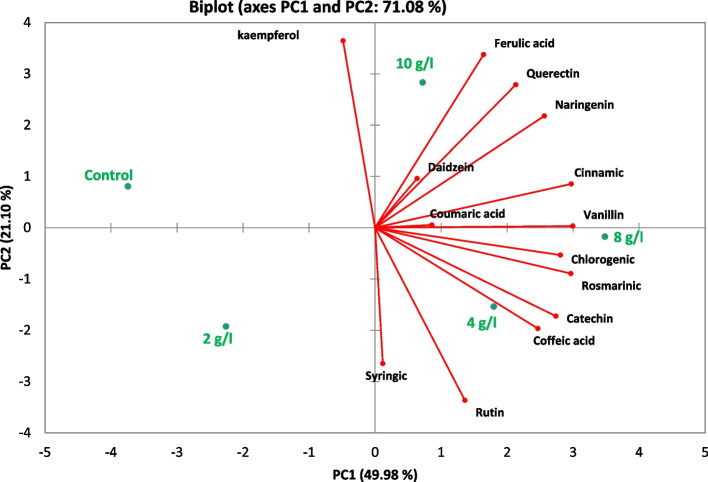
Principal component analysis of phenolic and flavonoids compounds measured at control and different DPS concentrations of *L. arabicus* L callus (2, 4, 8 and 10 g/l)

As represented in Fig. 10, PCA was performed to reveal the interrelationships of phenolic and flavonoid fractions in the control and different DPS sets and to confirm the variations among the aforementioned fractions. The results showed that the first four components (PCs) with eigenvalues ≥ 1 accounted for 100% of the total variation. The first PC accounted for 48.41% of the total variation in the data and was significantly correlated with rosmarinic acid, vanillin, catechin, chlorogenic acid, cinnamic acid, caffeic acid and naringenin in descending order. PC2 explained 21.1% of the total variation and was influenced mainly by kaempferol, ferulic acid and quercetin. PC3 accounted for 18% of the total variation and was strongly correlated with syringic acid and coumaric acid. PC4 accounted for 10.9% of the total variation and was correlated mainly with daidzein and rutin. PC4 accounted for only 4.64% of the total variation and had no noteworthy correlation with any of the measured traits. The PCA biplot of the first two PCs accounted for 44.11% of the overall variance.

## Discussion

Among natural elicitors, plant extracts are good sources of several phytochemicals that can elicit and boost plant growth and secondary mechanisms. The DPS was phytochemically characterized to screen its components and interpret its promoting impact. The eliciting trait of DPS could be attributed to its high macronutrients (N, P, K, Ca and Mg) and antioxidants contents. Likewise, previous results reported a rich elemental composition of date seed powder attaining high amounts of Ca, K and Mg [30]. Furthermore, high levels of antioxidant compounds including phenolics, flavonoids, carotenoids and sterols were previously demonstrated in DPS [17, 30]. Elicitation strategies usually rely on triggering the expression of genes involved in the plant stress response via the activation of specific receptors in the plasma membrane [31].

The results of GC analysis revealed the presence of a wide range of compounds that might be responsible for the high antioxidant potential of the DPS (Table 1, Fig. S1). For instance, HMF is the most abundant compound with a maximum peak area. HMF was reported to have crucial biological roles as an antioxidant, combating oxidative injury [32–34]. Like other fruits and vegetables, dates are known to produce HMF and other furan derivatives, probably from sugar dehydration [35]. The second major constituent was DDMP, which was identified as a strong antioxidant with free radical scavenging potential and wide availability in various plants [36]. Hence, date seeds are regarded as crucial sources of antioxidants and many phenolic compounds that are found in 65 to 78% of seeds [37].

Typically, adding elicitors to in vitro cultures improved cell growth and activated the production of secondary metabolites by switching primary metabolism as a defense response [38]. In this context, the present research investigated the ability of DPS powder to act as a natural elicitor. These findings are directly associated with the rich mineral composition of DPS (N, P, K, Ca and Mg) and elevated antioxidants that could boost callus metabolic processes and biomass. It was reported that the level of minerals, such as Ca, K, Si, and Mg, in date seed powder played a considerable role in the optimization of callus growth [30]. Furthermore, the addition of organic nitrogenous compounds (such as D-alanine N-propargyloxycarbonyl-isohexyl ester) could serve as a primary and rapid source for improved growth and surpasses the utilization of inorganic nitrogen sources [39]. Similarly, Akhtar et al. [40] reported that organic N sources and antioxidants reduced the oxidative browning of shoots and improved callus induction in *Rosa centifolia* during in vitro propagation. Thus, the distinct representation of bioactive components in DPS (Table 1) could be linked directly to the increase in callus biomass, particularly after 8 g/l treatment.

The measured total phenolic and flavonoid contents were significantly enhanced by DPS supplementation, where the favorable dose was 8 g/l. Phenolics play distinct roles in cell division, regulation of hormonal actions and nutrient mineralization [7]. Similarly, various elicitors, such as yeast pectin and salicylic acid, were utilized to increase total phenol and flavonoid contents and scale up the synthesis of nutraceutical compounds [41, 42]. The enhanced synthesis of phenolic compounds in callus cultures might be correlated with the triggered mitochondrial activity [43].

As a direct consequence of DPS addition in culture media, a distinct elevation in TAC and ASA contents was observed compared to the non-treated control. The present findings indicated that high TAC in the culture media was associated with increased cell proliferation in plant tissue cultures, as proven by the strong Pearson correlation (*p* < 0.05) (Fig. 8). Antioxidants can protect cells from oxidative stress and maintain cell viability, leading to enhanced cell division and tissue growth [44]. The increased TAC is strongly related to the antioxidant activity of the recognized HMF in DPS characterization (Table 1), suggesting the enhancement of medicinal and biological properties of the produced *L. arabicus* L callus treated with DPS.

The detected levels of MDA and H_2_O_2_ were considered signs of oxidative stress after the application of a higher DPS dose (10 g/l) in *L. arabicus* callus, while lower DPS doses accumulated normal levels relative to the control. Currently, MDA is a by-product of lipid peroxidation and refers to the stress intensity in plants [45]. The high dose of DPS offers increased availability of its bioactive components, such as furan derivatives, which might cause stressful conditions and high production of reactive oxygen species. However, the increase in stress markers could be linked to the enhanced level of antioxidants, including ASA, which might slightly relieve ROS and ameliorate toxicity. A principal proof was obtained by the negative Pearson correlation (*p* < 0.05) between the MDA contents and callus fresh and dry biomasses, secondary metabolites (phenolics and flavonoids) and TAC. Thus, the positive eliciting effect of DPS powder is a dose dependent application. In this context, optimization of the elicitor concentration is needed, as high levels can induce a hypersensitive response, leading to cell death [41].

The changes in the gene expression level of *PAL*, the prime enzyme in the phenylpropanoid pathway, significantly increased, with a maximum increase occurring at 8 g/l DPS compared to the control. PAL activation could be an indicator of the promotion of secondary metabolism as a direct shift from primary metabolism [46]. Thus, the aforementioned increase in phenolic and flavonoid levels by DPS application demonstrated the induction of phenylpropanoid pathways, which was evident by the overexpression of *PAL*. These findings were supported by the strong Pearson correlation (*p* < 0.05) shown in Fig. 8. To confirm the improvement in flavonoid biosynthesis after DPS treatment, the relative expression of several genes encoding enzymes mainly involoved in flavonoid synthesis was evaluated. The present results (Fig. 7) showed that the expression levels of the *CHS, CHI* and *FLS* were significantly upregulated in DPS-treated calli compared to the control, with the highest fold change in calli exposed to 8 g/l DPS. Likewise, chitosan-elicited *Isatis tinctoria* L. hairy root cultures attained elevated expression levels of *PAL, CHS* and *CHI* genes, which directly resulted in an increase in the total flavonoid content [47].

Moreover, our results revealed significant enhancements in *DXR* expression levels in calli treated with DPS in comparison to the control, with the highest fold change at 8 g/l DPS. The increased transcript levels of the *DXR* gene could account for the increased level of its functional protein and enzyme activity in *L. arabicus* callus. *DXR* is defined as an eliciting-responsive gene that widely contributes to normal plant growth and development boosting stress tolerance [48]. Therefore, this distinct upregulation of the *DXR* gene by DPS as an eliciting treatment could present evidence for activated terpenoid biosynthesis.

The phenolic acid and flavonoid contents of the treated calli were quantitatively analyzed using HPLC. The cinnamic acid derivatives, including chlorogenic and caffeic acid, were enhanced after DPS treatments with the greatest increase percentages compared to the control. They have remarkable biological properties, such as antioxidant, antibacterial, anticancer and anti-inflammatory properties [49, 50]. Chlorogenic and caffeic acids are produced by the shikimic acid pathway through aerobic respiration [51]. Their elevated biosynthesis and accumulation can be attributed to the elicited *PAL* gene expression. In this regard, the boosted activity of PAL was previously reported to be the main target of chlorogenic acid overproduction in *Eucommia ulmoides* [52]*.* Furthermore, rosmarinic acid level was highly increased as a key advantage of DPS treatment due to its known antimicrobial, anti-inflammatory and antimutagenic effects in both the pharmaceutical and cosmetic industries [53]. In addition, vanillin and ferulic acid levels were significantly induced in 8 g/l DPS-treated calli. Vanillin is known to have a principal therapeutic potential. The vanillin is known to be synthesized by ferulic acid decarboxylation; where enhanced ferulic acid level can be directed for vanillin production [54]. Therefore, these improvements in phenolic acid production are considerable proof of the potent eliciting ability of DPS.

The flavonoid components, including catechin, quercetin, rutin and naringenin, were highly increased by DPS application compared to the control. Various studies have revealed the vital role of catechin, quercetin and rutin as therapeutic agents that exhibit antioxidant, anti-inflammatory and anticarcinogenic activities [55]. The current DPS application indicated that the increased expression of *CHS, CHI* and *FLS* directly contributed to the upregulation of flavanols (catechin, quercetin and rutin) and flavanones (naringenin). Similarly, the overproduction of quercetin and rutin in *Delonix elata* callus was recorded by nanoparticles elicitation due to the induction of physiological and biochemical pathways [56]. However, the addition of the DPS-recommended dose (8 g/l) significantly decreased the kaempferol content relative to the control. The present findings may point to its potent utilization for the synthesis of key metabolites or a direct switch in their biosynthetic routes. It has been reported that kaempferol can serve as a precursor for integration into the production of the vital respiratory cofactor ubiquinone (coenzyme Q), benefiting metabolic processes and growth [57]. Thus, the highest secondary metabolites levels with potent nutraceutical values were successfully achieved by culture feeding with costless DPS powder in *L. arabicus.*

## Conclusion

The present study provides an unprecedented application of date palm seeds as a sustainable and costless plant elicitor. The phytochemical characterization of DPS revealed distinct phytocomponents, accounting for its vital eliciting role. The DPS treatment induced growth and phenolic and flavonoid content in *L. arabicus* calli, leading to increased antioxidant activity. Interestingly, DPS promoted the overexpression of eliciting-responsive genes (*PAL*, *CHS*, *CHI*, *FLS* and *DXR*) which are involved in the phenylpropanoid, flavonoid and terpenoid synthesis pathways. Taken together, in vitro feeding with DPS could be utilized as a promising elicitor and nutrient source, targeting the production of phenolics and flavonoids in *L. arabicus* culture. Therefore, further innovative approaches dealing with DPS need to be applied to define its superior biological and pharmacological characteristics.

### Supplementary Information

**Additional file 1: Fig. S1.** GC-MS chromatogram of the chemical constituents of date palm seeds (DPS). **Fig. S2.** HPLC chromatograms of phenolic and flavonoid compounds identified in A: control and DPS treated *L. arabicus* callus (B: 2 g/l, C: 4 g/l, D: 8 g/l and E: 10 g/l). **Fig. S3.** HPLC chromtogram for the standard polyphenols mixture. **Table S1.** Sequences of specific primers used in qRT-PCR. **Table S2.** Quantitative nutritional contents of DPS powder.

## Data Availability

Data is provided within the manuscript or supplementary information files.

## Abbreviations

ASA: Ascorbic acid
DPS: Date palm seeds
PAL: Phenylalanine ammonia lyase
CHS: Chalcone synthase
CHI: Chalcone isomerase
FLS: Flavonol synthase
DXR: Deoxyxylulose phosphate reductoisomerase
TAC: Total antioxidant capacity
H_2_O_2_: Hydrogen peroxide
MDA: Malondialdehyde
