# Supplementary material for: Upregulated synthesis and production of bioactive compounds in Lotus arabicus L. by in vitro feeding with dried powder of date palm seeds
Source: BMC Plant Biol. 2024 Mar 27;24:225. doi: 10.1186/s12870-024-04919-7 (PMC10976678; doi:10.1186/s12870-024-04919-7)
Supplement: Supplementary file 1 — Additional file 1: Fig. S1. GC-MS chromatogram of the chemical constituents of date palm seeds (DPS). Fig. S2. HPLC chromatograms of phenolic and flavonoid compounds identified in A: control and DPS treated L. arabicus callus (B: 2 g/l, C: 4 g/l, D: 8 g/l and E: 10 g/l). Fig. S3. HPLC chromtogram for the standard polyphenols mixture. Table S1. Sequences of specific primers used in qRT-PCR. Table S2. Quantitative nutritional contents of DPS powder. [file 12870_2024_4919_MOESM1_ESM.pdf]

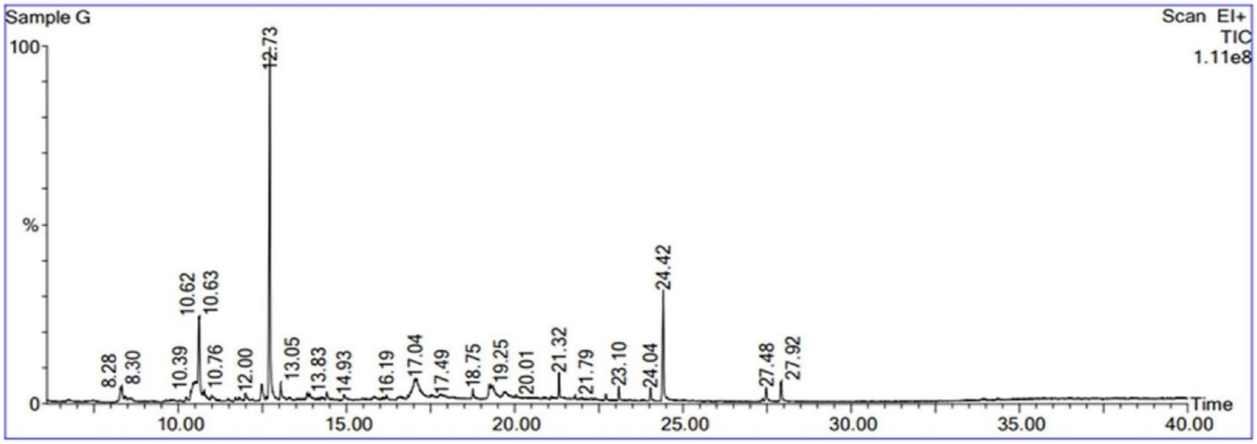

Fig. S1: GC-MS chromatogram of the chemical constituents of date palm seeds (DPS).

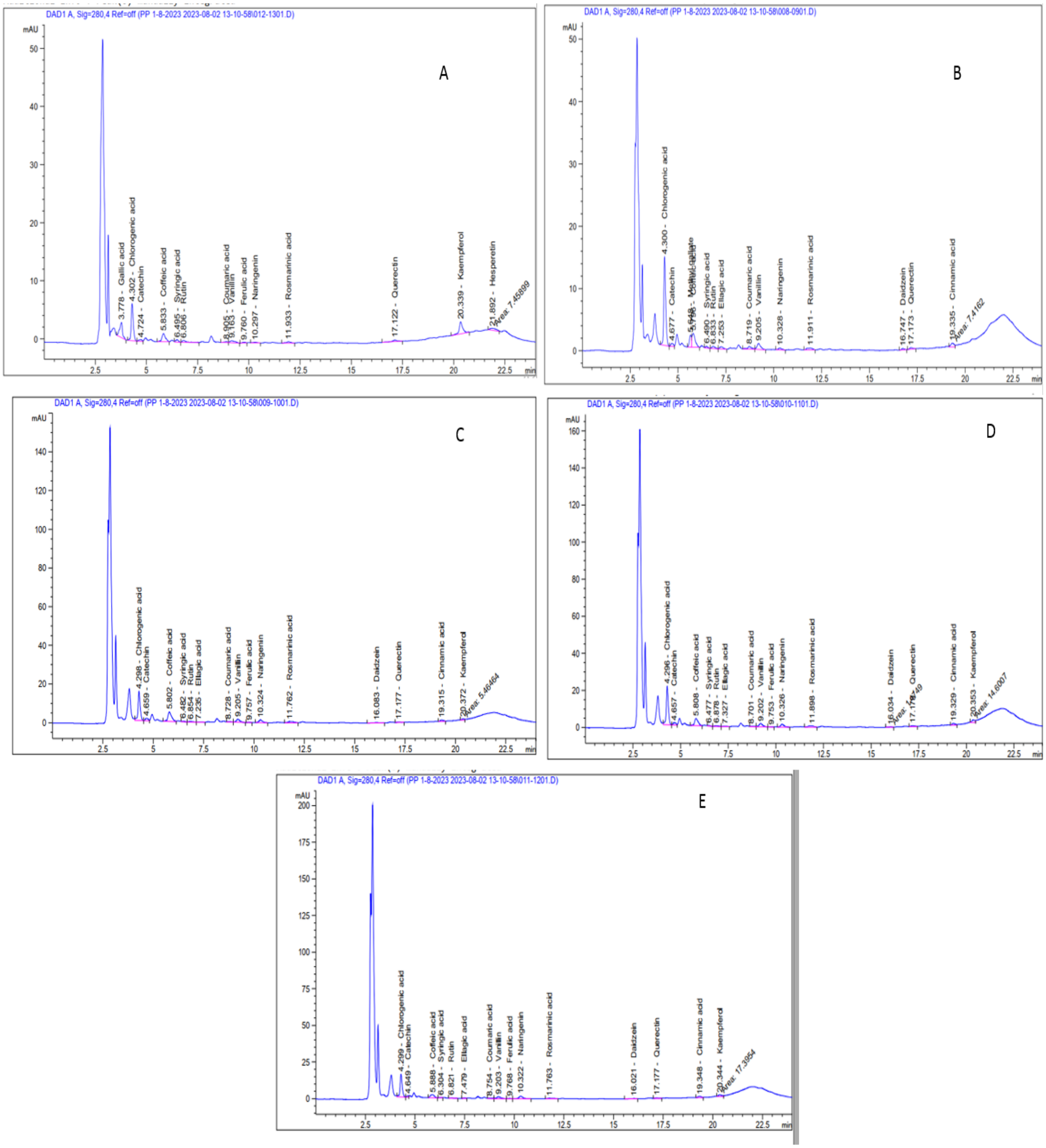

Fig. S2: HPLC chromatograms of phenolic and flavonoid compounds identified in A: control and DPS treated *L. arabicus* callus (B: 2 g/l, C: 4 g/l, D: 8 g/l and E: 10 g/l).

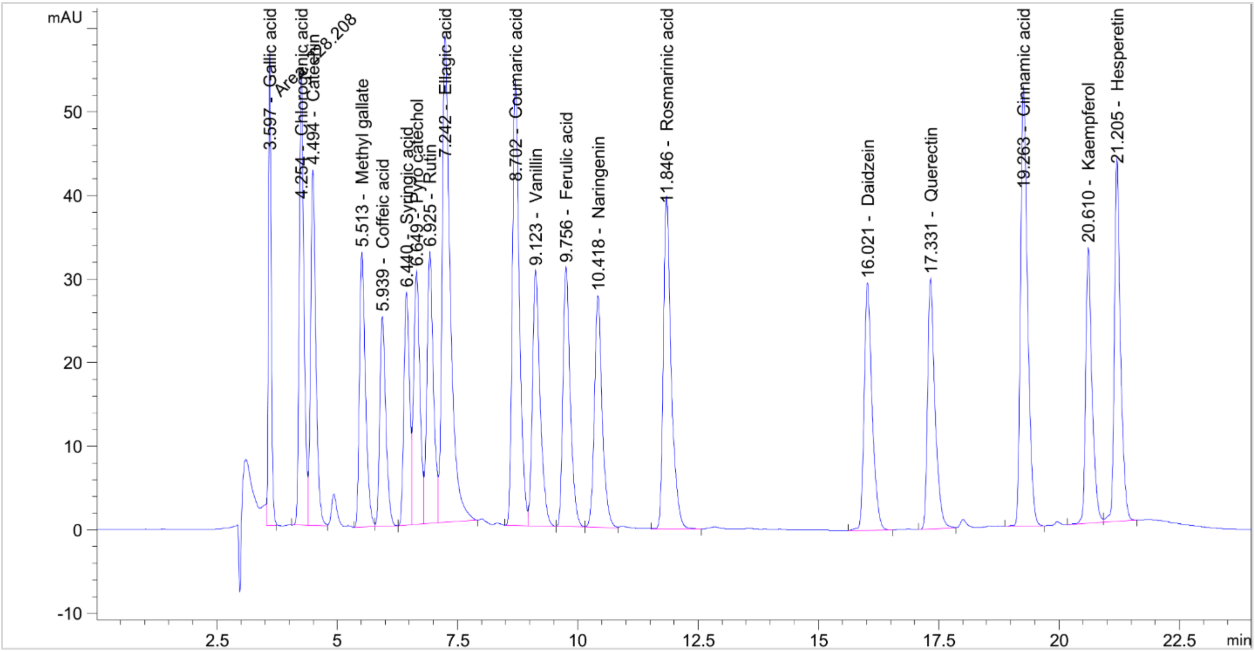

Fig. S3. HPLC chromtogram for the standard polyphenols mixture.

Table S1. Sequences of specific primers used in qRT-PCR

**Gene**	**Abbreviation**	**Direction**	**Sequences (5ˋ-3ˋ)**
Reference gene	*GAPDH*	F	AAGGTTATCAACGACAGGTTTG
		R	ATACCCTTAAGCTTGCCTTCTG
Phenylalanine ammonia lyase	*PAL*	F	GCAAGGAAAGCCCGAGTTTAC
		R	GGACCTTTTTGGCTACTTGGC
Chalcone synthase	*CHS*	F	CCCGATTACTATTTTCGGATCAC
		R	CGAGTGAATCAAGGTGAGTGTC
Chalcone isomerase	*CHI*	F	TGGTGGCCTAGACAACGATGAGTT
		R	TCACACTCCCAACTTGGTTTCCCT
Flavonol synthase	*FLS*	F	TTAAAGGAAGGTCTCGGTGGCGAA
		R	TCATTGGTGACGATGAGTGCGAGT
Deoxy-D-xylulose 5-phosphate reductoisomerase	*DXR*	F	TTGGTTGCTGAGCTAAAAGAAG
		R	CTTGTTGAAAAGTGTGGCAGAG

Table S2. Quantitative nutritional contents of DPS powder.

Parameters	Results	Unit
**N ions**	4.0±0.4	mg/g DM
**P ions**	8.3±0.4	mg/g DM
**K ions**	1.3±0.3	mg/g DM
**Ca ions**	1.2±0.1	mg/g DM
**Mg ions**	0.2±0.1	mg/g DM
**Total phenolic contents**	55.1 ± 8.1	mg/g DM
**Total flavonoid contents**	3.4 ± 0.5	mg/g DM
**Total antioxidant activity**	20.8 ± 0.6	mg ASA/g DM
